# Evaluation of sports training methods and technical characteristics based on multi-dimensional driving fuzzy intelligent computing

**DOI:** 10.1371/journal.pone.0316200

**Published:** 2025-06-02

**Authors:** Qiong Wu, Yi Sun, Lei Gao, Wanxing Yin

**Affiliations:** 1 College of physical education, China Three Gorges University, Yichang, Hubei, China; 2 Graduate School, Philippine Christian University, Malate, Manila, Philippines; 3 School of Physical Education, Da Lian University, DaLian, Liaoning, China; 4 School of Sports Training, Wuhan Sports University, Wuhan, Hubei China; Ningbo University, CHINA

## Abstract

To improve the competitive state of badminton athletes and summarize the technical characteristics of badminton players, this paper introduces multi-dimensional fuzzy removal intelligent computing. Taking 120 badminton students from a sports school as data samples, the sports images of athletes are collected, the images are enhanced using histogram equalization, and then the fuzzy clustering algorithm is used to analyze the characteristics of the pictures. The following results were obtained from the analysis of the understanding degree of motion decomposition, the analysis of the lasting effect, the study of the number of repetitions, and the analysis of the simulation results: The degree of understanding was 17.75% higher than that of traditional training methods; the effect was better than that of conventional training methods; the traditional training method had a small number of action repetitions; the performance of boys and girls in the temporary mock exam would be related to different training methods. Therefore, this paper had practical significance for this research, to help promote such academic and give reference. At the same time, most optimization problems needed to comprehensively consider many factors, so multi-objective optimization algorithms became a hot spot in academic research.

## 1. Introduction

In recent years, the research on data mining methods based on intelligent computing has made great progress, and the application of machine learning and other technologies has improved and enhanced the performance and efficiency of clustering mining to a certain extent. There is no doubt that the technical characteristics and training methods of physical education play an important role in physical education. This paper explored the technical characteristics of the multi-dimensional driven fuzzy intelligent algorithm, to make its research more systematic, comprehensive, complete, and scientific. It played a positive role in the improvement of athletes’ competitive level, the enhancement of athletes’ competitive level, the extension of athletes’ life span, and the reduction of athletes’ sports injuries.

Through scientific training methods, athletes can more effectively improve strength, endurance, speed, and flexibility, and optimize their physical fitness. At the same time, advanced technical means, such as video analysis and biomechanical evaluation, can help athletes identify and improve technical movements to ensure the safety and effectiveness of sports. By combining these methods and technologies, coaches can develop personalized training plans to promote the comprehensive development of athletes and ultimately achieve higher levels of competitive performance. Tabata Izumi found that badminton players used high-intensity interval sports training methods to improve their sports performance. He reviewed one of the most effective training methods from the perspective of sports energetics and also summarized previous studies on the metabolic profile and effects of sports training [1]. Fadde Peter J found that expert performers in many sports used excellent cognitive skills to identify the pattern of the opponent’s actions, to start quick reaction selection and exercise execution. Researchers developed technologies and methods to measure cognitive skills. These technologies and methods could also be used to improve these skills [2]. Ivanenko Stanislav believed that the existing training could not be used to acquire knowledge and skills in swimming or even learn the main sports way of badminton. The theoretical knowledge and practical skills of badminton players were satisfactory [3]. Kerr Zachary Y believed that badminton trainers were an important source of data collection to help generate the largest data set of college and high school sports injuries. These data helped each sport and policy committee formulate an agreement aimed at improving sports safety [4]. Zhang Jie found that with the increase of sports injury probability in sports training or competition, trunk support strength training was given more significance, including functional training and rehabilitation training. There were more and more research on trunk support strength training, but the results were not satisfactory [5]. Suchomel Timothy J believed that future research should explore how to better enforce eccentric and variable resistance under different training methods and how initial strength affected the performance of badminton players under different training methods [6]. Newcombe Daniel J systematically commented on the influence of technology and cognition in sports and concluded that constraint-oriented skills could obtain better skills [7]. Ramirez-Campillo Rodrigo believed that men’s and women’s volleyball players of different ages could improve VJH (Vertical Jump Height) through programs with relatively low volume and frequency, and found that PJT (Paroxysmal Junctional Tachycardia) seemed to effectively improve the VJH of volleyball players [8]. These sports training techniques have certain merits, but with the development of intelligent algorithms, they can be used to more accurately monitor athletes’ techniques and fatigue.

Intelligent algorithms play a vital role in sports training. Through data analysis and pattern recognition, they can monitor athletes’ performance in real-time, identify potential technical flaws and physical fatigue, and develop personalized training plans. In addition, intelligent algorithms can also help coaches and athletes make more scientific decisions and improve training efficiency and sports performance by simulating and predicting the effects of different training programs. Xu combined deep learning and the exploration of the relationship between ACL force and ankle joint movement patterns to develop a high-precision and easy-to-implement ACL force prediction model. The proposed ACL dynamic load force prediction model has good generalization ability and superior performance in terms of high accuracy [9]. Li Tuojian found that the application of intelligent recognition sports management systems in athlete sports management systems could effectively improve the quality of training and competition. At present, the technical bottleneck of most motion management systems is in the motion recognition classification module [10]. To accurately, quantitatively, and effectively measure and analyze the special movements of badminton players, Liang Bo developed a set of intelligent management systems for winter sports [11]. Liu Yuzhong believed that the main purpose of various methods for evaluating athlete feature recognition was to monitor the current health status of athletes, to provide some feedback on individual training quality. Based on deep learning and convolutional neural networks, the athlete target recognition was studied, which was a feature vector extraction method based on zero curvature [12]. Li Bin believed that intelligent computing had a positive impact on sports training and was conducive to the coordinated development of the sports industry and the realization of a sports power [13]. Song Hesheng discussed the complex algorithm of key motion data and extracted the accuracy of lack of training [14]. Nadeem Amir believed that the use of visual sensors to identify human activities was a challenging problem because there were changes in lighting conditions and complex movements during the monitoring of sports and fitness exercises [15]. These studies show that the application of intelligent computing has a positive role, but there are still some problems.

To understand the technical characteristics of badminton players and improve their competitive state, this paper collected data from some athletes, extracted their badminton sports characteristics using intelligent algorithms, and analyzed the athletes’ technical improvement using fuzzy intelligent computing. The results show that the algorithm in this paper can improve the training results of male and female athletes.

## 2 Methods

Experiment description: Based on multi-dimensional driving fuzzy intelligent calculation, this paper conducts simulation experiment analysis on sports training methods and technical characteristics, and selects 120 badminton students from a vocational sports school as data samples. Among them, 60 students are using traditional training methods and 60 students using new training methods. The ratio of men and women in traditional training methods is 46:14, and that in new training methods is 35:25.

The experiment is carried out from the analysis of motion decomposition understanding, continuous effect, repetition, and simulated performance. Table 1 shows the analysis of the ways for students to acquire sports knowledge:

**Table 1:** Analysis of students’ access to sports knowledge.

	Number of people	Boys/%	Number of people	Girls/%
Curriculum	14	17.3	4	10.3
Network	47	58	12	30.8
Book	9	11.1	13	33.3
Sports team	11	13.6	10	25.6

### 2.1 Graphics enhancement

The image processing algorithm is the core part of image processing technology, which is mainly used to enhance, analyze, and transform images. On this basis, the collected data is processed and the data is processed. Histogram equalization (HE) is a method that uses the statistical data of the histogram to modify it. It can effectively process the histogram distribution of the original image so that each gray level has a uniform probability distribution. It can automatically improve the contrast of the overall image by adjusting the dynamic range of the grayscale value of the image so that the image has a large contrast and most of the details can be seen. The classic histogram analysis method is as follows: the input histogram is described by H(p); the input grayscale range is [p0, pk], and the goal is to find a monotonic pixel brightness transformation q= T (p) so that the output histogram G(p) is uniform in the entire output brightness range [p0, pk]. The histogram can be considered as a discrete probability density function:


$$\overset{\text{k}}{\underset{\text{i}=0}{\sum }}\text{G}\left({\text{q}}_{\text{i}}\right)=\overset{\text{k}}{\underset{\text{i}=0}{\sum }}\text{H}\left({\text{p}}_{\text{i}}\right)$$ (1)

The sum in formula (1) can be understood as the accumulation of discrete probability density functions. Assuming that there are M rows and N columns of pixels in an image, the balanced histogram G(p) corresponds to the balanced discrete probability density function f, whose value is a constant:


$$\text{f}=\text{MN}/\left({\text{q}}_{\text{k}}-{\text{q}}_{0}\right)$$ (2)

Substituting the value of equation (2) into the left side of equation (1), we can get an accurate equalized histogram. At this time, equation (1) becomes:


$$\text{MN}\int_{{\text{q}}_{0}}^{\text{q}}\frac{1}{{\text{q}}_{\text{k}}-{\text{q}}_{0}}\text{ds}=\frac{\text{MN}\left(\text{q}-{\text{q}}_{0}\right)}{{\text{q}}_{\text{k}}-{\text{q}}_{0}}=\int_{{\text{p}}_{0}}^{\text{p}}\text{H}\left(\text{s}\right)\text{ds}$$ (3)

This gives the image brightness transformation T:


$$\text{q}=\text{T}\left(\text{p}\right)=\frac{{\text{q}}_{\text{k}}-{\text{q}}_{0}}{\text{MN}}\int_{{\text{p}}_{0}}^{\text{p}}\text{H}\left(\text{s}\right)\text{ds}+{\text{q}}_{0}$$ (4)

The integral in formula (4) is called the accumulated histogram, which is usually approximated by superposition, and the resulting histograms are not completely equal. For the discrete case, the approximation of the continuous pixel brightness conversion in formula (4) is as follows


$$\text{q}=\text{T}\left(\text{p}\right)=\frac{{\text{q}}_{\text{k}}-{\text{q}}_{0}}{\text{MN}}\int_{{\text{p}}_{0}}^{\text{p}}\text{H}\left(\text{s}\right)\Delta \text{s}+{\text{q}}_{0}$$ (5)

**Fig 1:**
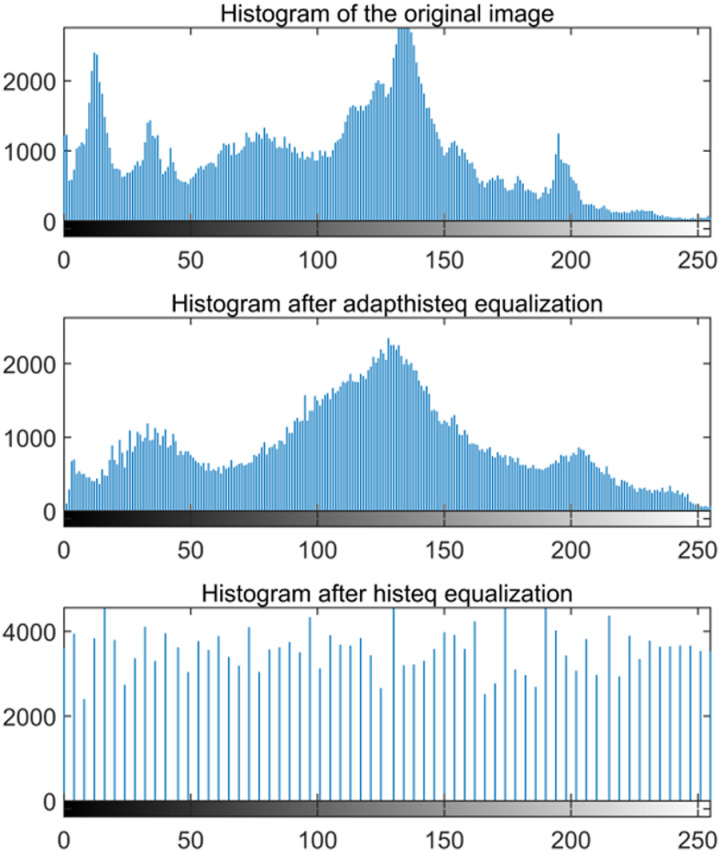
Histogram equalization.

This paper uses the histogram equalization image enhancement algorithm for enhancement. The image before and after the histogram equalization image enhancement is shown in Fig 1.

### 2.2 Intelligent decision-making sports training

**Fig 2:**
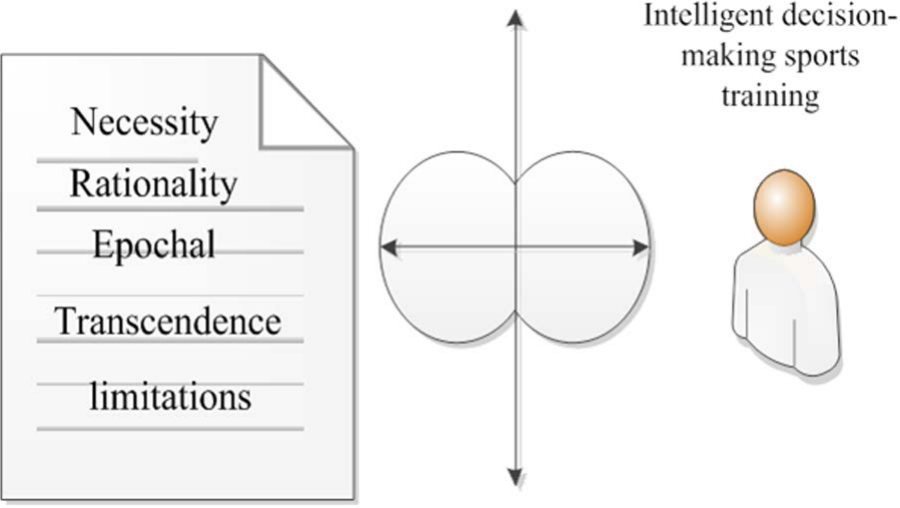
Intelligent decision-making exercise training.

Intelligent decision-making is a synthesis of knowledge and skills, which is a combination of effective cognition of objective things and effective implementation in life [16]. Intelligence is a stable psychological feature, which provides a person with cognitive activities, including five basic elements: perception, memory, imagination, thinking ability, and attention. The ability includes organizational ability, planning ability, practical ability, adaptability, and creativity. Sports intelligence belongs to the category of intelligence and is an important aspect of athletes’ comprehensive quality. Sports intelligence training refers to the training and cultivation of various elements of sports intelligence in a planned and step-by-step manner according to the requirements of modern sports teaching, and the organic combination of them to improve the training of athletes, as shown in Fig 2.

1) Necessity

Competitive ability refers to the ability of athletes in the competition, including physical skills, tactical ability, sports intelligence, psychological ability, and other forms of expression and functions, which are fully reflected in the whole process of the competition. In traditional training, skills training, tactics training, and psychological training are emphasized, but there are deviations in intelligence training. In the system theory, the famous barrel theory believes that the volume of a barrel composed of several boards is not determined by the longest board but by the shortest board. The lack of athletes’ physical quality or ability would hurt the competition. Therefore, it is necessary to strengthen the cultivation of athletes’ intelligence.

2) Rationality

Intelligent training is an effective way to solve the contradiction between learning and training, promote the all-round development of athletes, and integrate them into society. Especially for young athletes, their physical development is fast and their thinking is active. The physiological and psychological changes are relatively large, and this is a critical period for rapid intellectual development. Many people neglect the cultivation of athletes’ intelligence and only pay attention to the cultivation of skills, achievements, ideas, culture, and other aspects, which leads to ideological problems. The low level of culture delays the progress and development of sports technology. The demand for athletes’ self-development determines the all-round comprehensive education and intensive intellectual training for athletes. The career of athletes has its limits. For most athletes, whether they can engage in a job in the future is mainly affected by their intellectual development.

3) Epochal

With the development of physical education, has entered a new stage of development. Informatization training is to make full use of information and the law of information to conduct optimal information control on the sports training process in scientific training so that the whole training process becomes a whole. To achieve information training, it is necessary to build a complete modern information system. At the same time, the information literacy of coaches and athletes should be strengthened. Information ability is based on the level of knowledge, that is the level of intelligence. It is an important content of information technology training to improve the knowledge and cultural accomplishment of coaches and athletes to enhance their intelligence.

4) Transcendence

The current sports training regards athletes as passive and mechanical, and the essential feature of sports intelligence is its high initiative. The goal of sports training is to develop people’s competitive ability as well as their intelligence. Therefore, the research and development of intelligence should be taken as the starting point of training. Many teachers neglect the research, development, and training of intelligence, which inevitably leads to mistakes and blind spots in training.

5) Limitations

At present, there is no in-depth theoretical exploration of the research of athletes’ intelligence, and there is no appropriate method for research. It is embodied in the fuzziness of the structure, constituent elements, interrelationship, and essential characteristics of athletes’ intelligence. At the micro level, the special tools and detection means of athletes’ intelligence tests are still blank. In intelligence training, due to the lack of theoretical knowledge, coaches and athletes doubt intelligence training, which is the biggest obstacle in intelligence training.

### 3. Related Algorithms of Fuzzy Intelligent Computing

(1) Color similarity measurement

Color characteristics can select three channels from any color space, and their eigenvalues can be represented by$I_{1}$, $I_{2}$and$I_{3}$. The color similarity index of pixel (x,y) is as follows:


$$S_{i}^{c}(x,y)=1-\frac{\left|I_{i}^{c}(x,y)-I_{i}^{b}(x,y)\right|}{1}$$ (6)

In the formula, $I_{i}^{c}$and$I_{i}^{b}$ are foreground pixel values and back scene values, respectively.

(2) Texture similarity measurement

An intelligent computing algorithm is used to represent texture features, which not only retains the excellent performance of the algorithm but also has a strong anti-interference ability to noise and can effectively improve the accuracy of shadow detection. The texture similarity (x, y) of graph pixels points is expressed as follows:


$$S_{i}^{t}(x,y)=1-\frac{\left|t_{j}^{c}(x,y)-t_{j}^{b}(x,y)\right|}{1}$$ (7)

In the formula, $t_{j}^{c}$and$t_{j}^{b}$ are the foreground pixel texture values and the background pixel texture values, respectively.

(3) Fuzzy clustering algorithm

When choosing the fuzzy clustering method, two problems must be considered: One is that the features of the target object can accurately describe the moving posture, and the other is that the fuzzy features of the moving position and the invalid pixel area cannot be completely consistent. The action characteristics are analyzed using a fuzzy clustering algorithm.

d(x,y) represent the pixel, and an empty moving rectangle is used to measure the temporal dominance matrix of the image. The formula is as follows:


$$m_{d}=\frac{1}{n}\sum_{1}\left[d(x,y)-\bar{m}\right]$$ (8)

Among them, $m_{d}$represents a fourth-order matrix, and n is the total number of pixels covered during the motion; $\bar{m}$represents the average gray value of each pixel, and its function is expressed as follows:


$$m_{d}=\frac{1}{n}\sum_{1}d(x,y)$$ (9)

Due to the complexity of matrix operation and its insensitivity to a normal distribution, the matrix is decomposed into a second-order matrix to identify the ambiguity of the difference matrix in the time domain.


$$m_{d}=\frac{1}{n}\sum_{1}{\left[d(x,y)-\bar{m}\right]}^{2}$$ (10)

It is assumed that$u_{mr}(x,y)$ is the background membership function of sports video image fuzzy attribute and $u_{nr}(x,y)$is the foreground membership number.


$$u_{mr}(x,y)=s(x,y,a,b)=\left\{\begin{array}{l} 0\\ 1\\ \frac{2m_{d}^{2}-2a}{b-a}\\ 1-\frac{2m_{d}^{2}-2a}{b-a} \end{array}\right\}$$ (11)

$$u_{mr}(x,y)=1-u_{nr}(x,y)$$ (12)

Among them, a and b are weight parameters.

## 4. Simulation experiment of traditional training methods and motion training methods of multi-dimensional driving fuzzy intelligent computing

(1) Analysis of understanding of action decomposition

**Fig 3:**
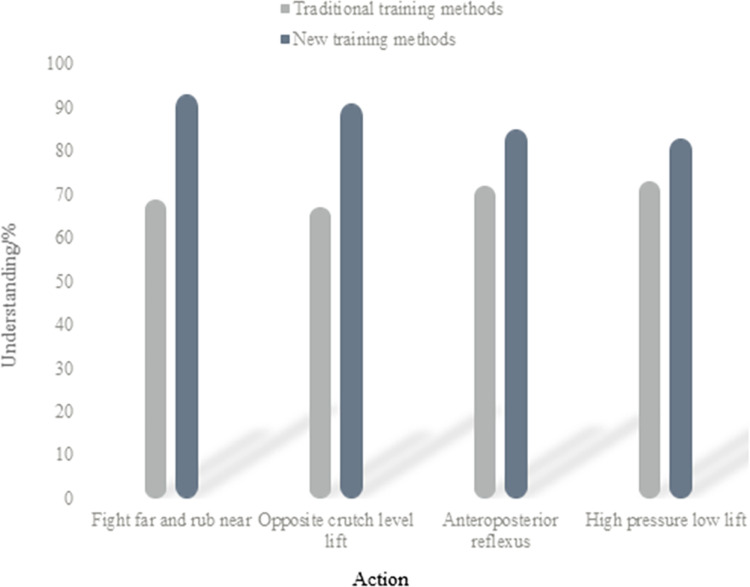
Analysis of action decomposition understanding in traditional and new training methods.

By taking badminton as the reference object, this paper analyzes the understanding of four movements under two training methods, as shown in Fig 3:

Fig 3 shows that traditional training methods in badminton initially decline and then rise, while new training methods consistently decline. Under the traditional training mode, students’ understanding of movement decomposition is as follows: “fight far and rub near” at 69%, “opposite crutch level lift” at 67%, “anteroposterior reflexes” at 72%, and “high pressure and low lift” at 73%. In contrast, under the new training mode, understanding improves significantly: “fight far and rub near” at 93%, “opposite crutch level lift” at 91%, “anteroposterior reflexes” at 85%, and “high pressure and low lift” at 83%. The new training method shows a 24% increase in understanding for both “fight far and rub near” and “opposite crutch level lift,” with increases of 13% for “anteroposterior reflexes” and 10% for “high pressure and low lift.” Overall, the average understanding under traditional methods is 70.25%, while new methods reach 88%, indicating a 17.75% improvement in understanding with the new training approach.

(2) Continuous effect analysis

**Fig 4:**
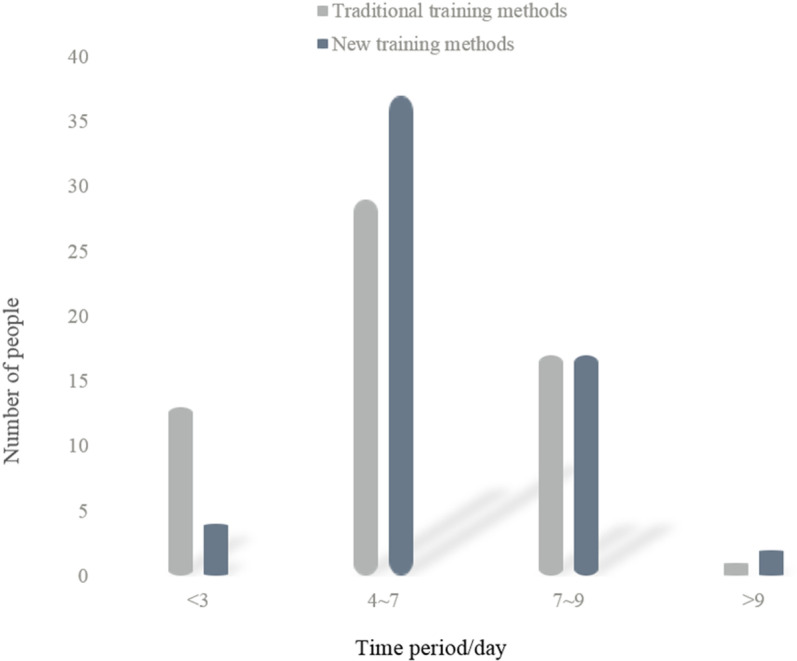
Period analysis of understanding the lasting effect of badminton decomposition.

Based on the results of experiment 1, the understanding and lasting effect of badminton decomposition action is analyzed, as shown in Fig 4:

It can be seen from Fig 4 that 13 people have understood badminton movements for less than 3 days in traditional training methods; 29 people understand the duration of 4–7 days; 17 people understand the duration of 7–9 days; one person understands the duration for more than 9 days. In the new training mode, 4 people understand badminton movements for less than 3 days; 37 people understand the duration of 4–7 days; 17 people understand the duration of 7–9 days; 2 people understand the duration for more than 9 days. It can be seen that in the traditional training mode, the number of people is mainly concentrated in 4–7 days, which is about 48.3% of the total number. In the new training mode, the number of people is also concentrated in 4–7 days, which is about 61.7% of the total number. It can also be seen from the distribution of the number of people that the number of people less than 3 days in the traditional training method is about 21.7% of the total number of people, while the number of people less than 3 days in the new training method is about 6.7% of the total number of people. Therefore, it is obvious that the new training method using multi-dimensional driven fuzzy intelligent computing is better than the traditional training method.

(3) Repetition analysis

**Fig 5:**
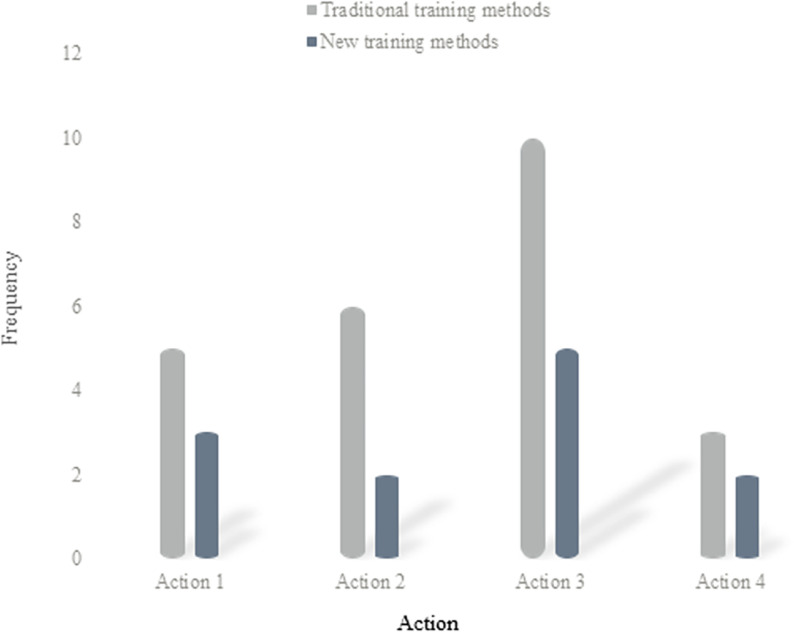
Analyze two training methods for repeating the next set of actions.

Fig 5 shows the analysis of the number of repetitions of a group of actions under the two training methods:

According to Fig 5, the number of repetitions of actions 1, 2, 3, and 4 in the traditional training mode is 5, 6, 10, and 3 respectively; under the new training mode, the number of repetitions of actions 1, 2, 3 and 4 is 3, 2, 5 and 2 respectively. It can be seen that for the same group of movements, the traditional training method has more repetitions than the new training method, which greatly reduces the training time and helps them overcome their unskilled movements.

Based on the above experimental analysis of badminton, students who use traditional training methods and new training methods that use multi-dimensional driving fuzzy intelligent computing are temporarily simulated, and the results of boys and girls are distinguished for more summary and analysis, as shown in experiments 4 and 5:

(4) Analysis of boys’ simulated performance

**Fig 6:**
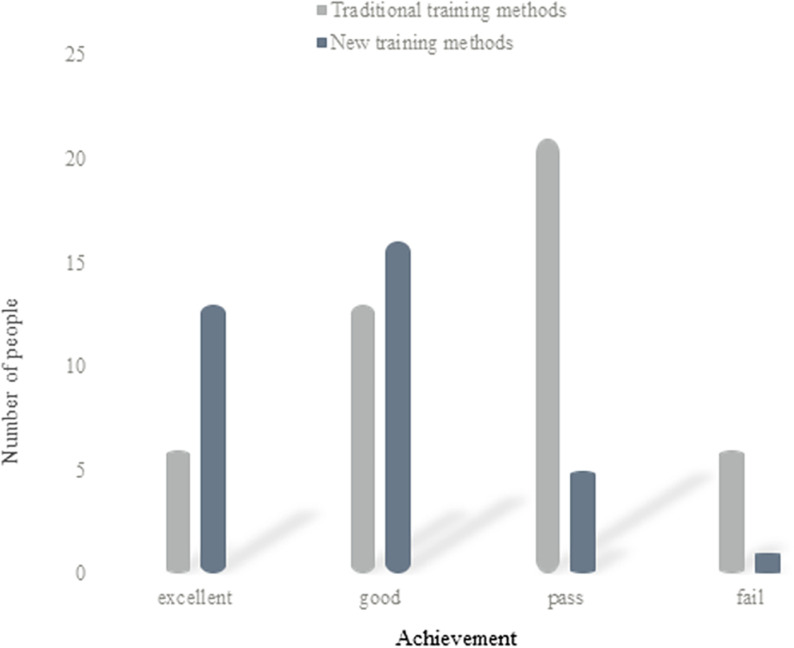
Analysis of boys’ simulation scores.

Fig 6 shows the analysis of boys’ simulated performance in two ways:

It can be concluded from Fig 6 that in the traditional training mode, there are 6 boys with excellent simulation results; there are 13 people with good simulation results; there are 21 persons who pass the simulation; a total of 6 people fail the simulation. Among them, the excellent rate is about 13%; the good rate is 28.3%; the passing rate is about 45.7%; the failing rate is about 13%. In the new training mode, there are 13 boys with excellent simulation results; there are 16 persons with good simulation results; there are 5 persons who pass the simulation; there is 1 person in total who fails the simulation. Among them, the excellent rate is about 37.1%; the good rate is 45.7%; the pass rate is about 14.3%; the fail rate is about 2.9%. According to the number of people, it can be calculated that in the traditional training mode, 40 boys pass the simulation test or above, which is about 87% of the total number. A total of 6 persons failed in their performance, which is about 13% of the total number. In the new training mode, there are 34 male students whose simulation results are above the pass, accounting for 97% of the total number. A total of 1 person fails the exam, which is about 3% of the total number. Therefore, it can be seen that the performance of the new training method using multidimensional-driven fuzzy intelligent computing is better than that of the traditional training method.

(5) Analysis of girls’ simulated performance

**Fig 7:**
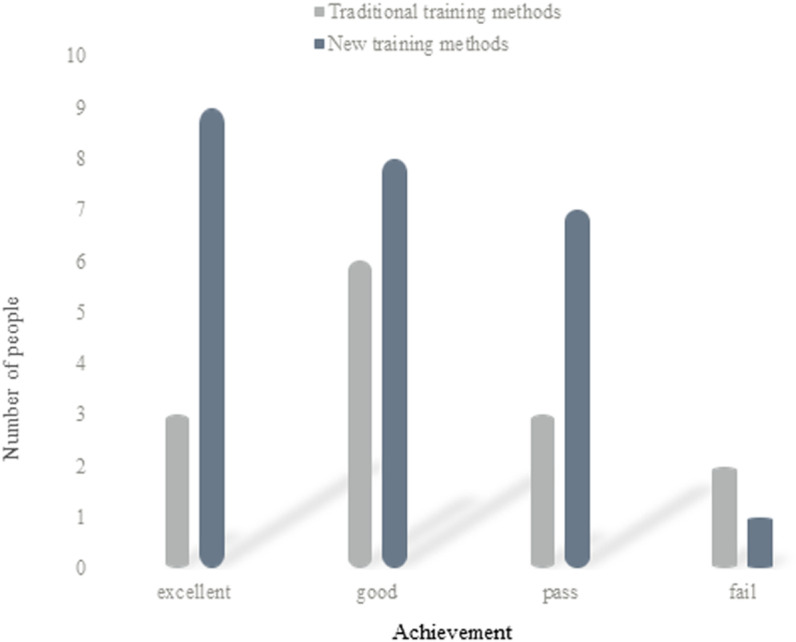
Analysis of girls’ simulation performance.

Fig 7 shows the analysis of the simulated performance of girls in two ways:

It can be concluded from Fig 7 that there are 3 female students with excellent simulation results in traditional training methods; there are 6 persons with good simulation results; there are 3 persons who pass the simulation; there are 2 persons who fail in the simulation. Among them, the excellent rate is about 21.4%; the good rate is 42.9%; the passing rate is about 21.4%; the failing rate is about 14.3%. In the new training mode, there are 9 girls with excellent simulation results; there are 8 persons with good simulation results; there are 7 persons who pass the simulation; there is 1 person in total who fails the simulation. Among them, the excellent rate is about 36%; the good rate is 32%; the passing rate is about 28%; the failing rate is about 4%. It can be calculated from the number of students that, in the traditional training mode, 12 girls pass the simulation test or above, which is about 86% of the total number. A total of 2 persons failed the examination, accounting for 14% of the total number. In the new training mode, there are 24 girls whose simulation results are above the pass level, accounting for 96% of the total number. A total of 1 person failed the exam, which is about 4% of the total number. It can be seen that the new training mode of multi-dimensional driving fuzzy intelligent calculation is better than the simulation of the traditional training mode for girls.

To sum up, this paper analyzes the sports training methods and technical characteristics based on multi-dimensional driving fuzzy intelligent computing and draws the following results from the above five experiments: The average understanding of action decomposition of the new training method in badminton is 17.75% higher than that of the traditional training method; the effect of the new training method is better than that of the traditional training method, which reduces the training time and the different performance of men and women’s simulation results.

## 5. Conclusions

Motion analysis is a very broad field covering many fields, such as computer vision, artificial intelligence, and model recognition. Cognitive computing is an intelligent computer system based on human knowledge. This paper analyzed the methods and technical characteristics of sports training through multi-dimensional driving fuzzy intelligent calculation and put forward corresponding methods for this purpose. At the same time, badminton, a national sport, was simulated and analyzed. It was concluded that this new training method had advantages over the traditional training method in many aspects. This paper conducted an in-depth analysis of sports training methods and technical characteristics through multi-dimensional driven fuzzy intelligent computing and took badminton as an example to conclude that the new training method is better than traditional training methods in understanding movement decomposition, improving training effects, and shortening training time. It has significant advantages in other aspects, but there are still certain limitations. Specifically, this study mainly focused on badminton, a single sport, and its applicability to other sports such as basketball, football, swimming, etc. has not been fully verified. Therefore, in the future, we will further expand the sample size and sports scope, not only increasing the sample size of badminton players but also introducing athletes from other sports to comprehensively verify the universal applicability and effectiveness of this new training method.

## Supporting information

S1 Data Article minimum data set. (XLSX)
